# A physiological comparison of the new—over 70 years of age—marathon record holder and his predecessor: A case report

**DOI:** 10.3389/fphys.2023.1122315

**Published:** 2023-02-13

**Authors:** Bas Van Hooren, Romuald Lepers

**Affiliations:** ^1^ NUTRIM School of Nutrition and Translational Research in Metabolism, Maastricht University Medical Centre, Department of Nutrition and Movement Sciences, Maastricht, Netherlands; ^2^ INSERM UMR1093, Cognition Action et Plasticité Sensorimotrice, Faculty of Sport Sciences, University of Bourgogne, Dijon, France

**Keywords:** aging, running, master athlete, oxygen consumption, case report, running economy, training characteristics

## Abstract

**Purpose:** This study assessed the body composition, cardiorespiratory fitness, fiber type and mitochondrial function, and training characteristics of a 71-year-old runner who broke the world record marathon of the men’s 70–74 age category and held several other world records. The values were compared to those of the previous world-record holder.

**Methods:** Body fat percentage was assessed using air-displacement plethysmography. $\dot{V}O_{2}\max$ , running economy, and maximum heart rate were measured during treadmill running. Muscle fiber typology and mitochondrial function were evaluated using a muscle biopsy.

**Results:** Body fat percentage was 13.5%, $\dot{V}O_{2}\max$ was 46.6 ml kg^−1^ min^−1^, and maximum heartrate was 160 beats∙min^-1^. At the marathon pace (14.5 km h^−1^), his running economy was 170.5 ml kg^−1^ km^−1^. The gas exchange threshold and respiratory compensation point occurred at 75.7% and 93.9% of the $\dot{V}O_{2}\max$ , i.e., 13 km h^−1^ and 15 km h^−1^, respectively. The oxygen uptake at the marathon pace corresponded to 88.5% of $\dot{V}O_{2}\max$ . Vastus lateralis fiber content was 90.3% type I and 9.7% type II. Average distance was 139 km∙w^−1^ in the year prior to the record.

**Conclusion:** The 71-year-old world-record holder marathon showed a relatively similar $\dot{V}O_{2}\max$ , lower percentage of $\dot{V}O_{2}\max$ at marathon pace, but a substantially better running economy than his predecessor. The better running economy may result from an almost double weekly training volume compared to the predecessor and a high type I fiber content. He trained every day in the last ∼1.5 years and achieved international performance in his age group category with a small (<5% per decade) age-related decline in marathon performance.

## 1 Introduction

Participation in distance running events such as marathons has risen in recent years, with this rise being primarily driven by the increased participation of master (>35 years) athletes (Willy and Paquette, 2019). Distance running performance is largely determined by three physiological factors, including the maximal oxygen uptake ( $\dot{V}O_{2}\max$ ), running economy, and lactate threshold (Joyner, 1991). While different combinations among these physiological determinants can lead to similar performances (Costill et al., 1973; Lucia et al., 2006), aging is associated with progressive reductions in cardiovascular and neuromuscular functioning that contribute to the decline in these physiological determinants and thus to the decrease in running performance with increasing age (Brisswalter and Nosaka, 2013; dos Anjos Souza et al., 2023; Ganse and Degens, 2021; Allen et al., 1985). For example, the maximum cardiorespiratory fitness as measured with the maximal oxygen uptake ( $\dot{V}O_{2}\max$ ) decreases by ∼0.5–1% per year from 30 years onwards (Fleg and Lakatta, 1988; Fuchi et al., 1989; Trappe et al., 1996), with this decrease being mainly due to lower cardiac output as a consequence of the reduction in the maximum heart rate (∼0.7 beats∙minute^-1^ per year (Tanaka et al., 2001)), and decrease in muscle mass (Fleg and Lakatta, 1988). Running economy at a fixed running speed has also been reported to decrease with aging in some (dos Anjos Souza et al., 2023; Pantoja et al., 2016) but not all (Allen et al., 1985; Quinn et al., 2011; Beck et al., 2016) studies, with this decrease being partly explained by reductions in tendon stiffness (Karamanidis and Arampatzis, 2006; Mademli and Arampatzis, 2008), that in turn decreases the mechanical efficiency of the muscle (Bohm et al., 2019). Finally, the metabolic rate at which the lactate threshold occurs has also been reported to decrease (Wiswell et al., 2000) or not change with increasing age (Allen et al., 1985). Since the decrease in $\dot{V}O_{2}\max$ with age is larger than the decrease in lactate threshold, the lactate threshold typically occurs at a higher percentage of $\dot{V}O_{2}\max$ (Allen et al., 1985; Wiswell et al., 2000).

The study of master athletes, and elite or world-class master athletes in particular, can provide essential insights into the ability of humans to maintain a high $\dot{V}O_{2}\max$ , lactate threshold, and running economy and thereby running performance with advancing age, and on the training methods to do so (Rittweger et al., 2009; Tanaka et al., 2011; Lepers and Stapley, 2016; Lazarus and Harridge, 2017). However, studies among world-class master athletes are rare. In the few studies performed to date, world-class endurance master athletes were found to exhibit a very high cardiorespiratory fitness as shown through a $\dot{V}O_{2}\max$ of 64.5 ml kg^−1^.min^−1^ at the age of 60 years (Lepers et al., 2020) and 46.9 and 50 ml kg^−1^.min^−1^ at the age of 70 (Robinson et al., 2019) and 75 years (Van Hooren et al., 2022), respectively. These values are higher than would be expected for their age as predicted from the 1% decline per year from 30 years onwards (Fleg and Lakatta, 1988). Similarly, these case studies showed that high-level masters marathoners (>60 years old) could sustain a very high fraction (91%–94%) of their $\dot{V}O_{2}\max$ up to ∼3 h (Robinson et al., 2019; Lepers et al., 2020), and exhibited comparable running economy compared to younger competitive individuals (Robinson et al., 2019).

On the 8th of May 2022, the marathon world record for the over 70 years category was broken by 4s (2:54:19 vs. 2:54:23 (h:min:s)). Robinson et al. (Robinson et al., 2019) published a case study that documented the physiological profile of the previous 70-year-old record holder who set the record in December 2018. Here, we report the physiological profile of the new world record holder, and compare it to that of the previous world record holder to gain insight into the physiological requirements for world-class performance in aging athletes. We hypothesize that the new world record holder would show a better running economy than his predecessor as the new world record holder completed a high training volume that in turn may improve running economy (Barnes and Kilding, 2014). We also hypothesized that the new record holder would have a similar or lower $\dot{V}O_{2}\max$ than his predecessor, because other studies have shown a tradeoff between running economy and $\dot{V}O_{2}\max$ (Fletcher et al., 2009; Shaw et al., 2015; Lannerstrom et al., 2021). Moreover, we also document the training characteristics of the athlete in an attempt to determine what attributes of training may have contributed to the exceptional performance. Such information may provide valuable insights to individuals seeking to optimize performance in aging athletes. Finally, we also explore potential mechanistic reasons for superior performance using a muscle biopsy collected from the athlete as part of previous studies (Grevendonk et al., 2021; McCrum et al., 2021). This technique made it possible to assess fiber type distribution, and *ex vivo* mitochondrial respiration capacity, and mitochondrial content.

## 2 Methods

### 2.1 Subject

A 71-year-old Dutch world-class Caucasian long-distance athlete with a height and weight of 181.0 cm and 64.8 kg participated in this study. His competition distances were half-marathons to ultramarathons, with a focus on marathons. He started running at the age of 36 years after being advised to pick up exercise by a physician to lower his blood cholesterol levels. His best running times are 16:42 min:sec at 5 km at the age of 54, 34:35 min:sec at 10 km at the age of 49, and 2:41:01 h:min:sec at the marathon at 54 years old. The athlete currently holds the world record for the 6-h run in the 55–59, 60–64, and 65–69 age categories. He recently got the world record marathon in 2:54:19 h:min:sec in the 70–74 age category (Supplemental Figure S1)

The athletes’ self-reported average weekly training distance in the year leading up to his world-record marathon was 139 km week^−1^, completed in seven sessions per week (Table 1). In this year (as well as the other years) he regularly performed running sessions of up to 2 h 30 min. He performed no interval sessions on the track, but instead performed two fartlek sessions per week. In these sessions, the athlete would do very variable duration intervals, but typically ranging from 1 min to 15 min. All intervals were performed at an estimated 90% of his maximum capacity, and he tried to keep short (e.g., <1 min) rest periods between the intervals. The other training sessions were low-intensity runs guided by the ability to talk easily while running. Often, these sessions were performed as part of his job as a trainer in which he joined the start-to-run groups. This resulted in two and sometimes even three training sessions being completed per day. In addition, the athlete typically performed two core stability (e.g., front and side planks, body weight squat) sessions per week during his warm-up and reported including numerous easy hill runs in most of his training sessions as a form of strength training. He trained about 50% on concrete/asphalt and 50% on country roads or forest tracks. The athlete tracked all his training sessions with a wristwatch that included a GPS and optical heart rate monitor, and this data was used to compute the weekly and yearly training volumes and average running speed for several years (Table 1), as well as the training intensity distribution (Supplementary File S1).

**TABLE 1:** Training volume per year obtained from GPS.

Year	Yearly distance (km)	Weekly distance (km)		Average speed (km∙h^−1^)
2022	2307 (up to the World record marathon on 8 May 2022)^a^	136		8.40
2021	7,216^a^	139		8.92
2020	4,791	92		8.80
2019	5,329	102		8.61
2018	5,645	109		9.14
2017	5,431	104		9.37
2016	5,019	97		9.36
2015	4,206	81		9.39

^a^ The athlete ran every single day of the year during this year.

The athlete volunteered for the study and was informed about its nature and aims and the associated risks and discomfort before giving his oral and written consent to participate in the investigation. Specific written informed consent was obtained from the individual for the publication of any potentially identifiable images or data included in this article. The protocol was in conformity with the Declaration of Helsinki and was approved by the Research Ethics Committee of Maastricht University (nr. FHML-REC2022100). All experiments except for the muscle biopsy were performed on the same day with anthropometrics being measured first, followed by running economy, and finally, $\dot{V}O_{2}\max$ .

### 2.2 Anthropometrics

The height and weight of the athlete were taken using a wall-mounted stadiometer (Seca^©^ 216 stadiometer Seca, Hamburg) with an accuracy of 0.1 cm and a calibrated scale included in the Pod Bod technology to the nearest 0.1 kg, respectively. Body fat percentage was assessed before the cardiorespiratory measurements using air-displacement plethysmography (Bod Pod, Life measurement) using a Siri-3 compartment model and predicted lung volume, as described previously (Plasqui et al., 2012; Van Hooren et al., 2022).

### 2.3 Laboratory running economy and (v) $\dot{V}O_{2}\max$ assessment

The running protocol for measuring the running economy, $\dot{V}O_{2}\max$ , and the corresponding running velocity at $\dot{V}O_{2}\max$ (v $\dot{V}O_{2}\max$ ) was approximately matched to the previous case study (Robinson et al., 2019) for comparison purposes. Briefly, the athlete first completed an 8-min warm-up at a fixed speed of 8 km h^−1^ to allow familiarization with treadmill running (Van Hooren et al., 2020). This was followed by five 4-min bouts at speeds of 12, 13, 14, 15, and 16 km h^−1^ to assess the running economy. The participant recovered between each stage in the form of standing or sitting and typically amounted to 2–3 min as determined by the athlete. Finally, after ∼ 5 minutes of rest, the athlete started an incremental running test to determine $\dot{V}O_{2}\max$ . The incremental test started at 10 km h^−1,^ and the speed was increased by 0.5 km h^−1^ every minute until volitional exhaustion. The start speed and increments were chosen to allow determination of ventilatory thresholds and, at the same time reach v $\dot{V}O_{2}\max$ within ∼12 min (Jamnick et al., 2018) based on an estimated $v\dot{V}O_{2}\max$ of 17 km h^−1^. The inclination of the treadmill was kept constant at 0% because it best matches the physiological effort of overground running at speeds up to at least 16 km h^−1^ (Miller et al., 2019).

Running economy and $v\dot{V}O_{2}\max$ assessments were performed on a motorized treadmill (Technogym, Excite 700, Italy), with respiratory gases being captured using an indirect calorimeter (Cortex Metalyzer 3B, Cortex Biophysik, Leipzig, Germany). Heart rate was monitored using a Polar H10 chest strap connected *via* Bluetooth to the Cortex software. The participant was instructed to run as if he was running outside and wore the same shoe type (but a new pair) used during his world record marathon performance (Asics Metaspeed Sky plus).

The rate of oxygen consumption ( $\dot{V}O_{2}$ ) and carbon dioxide ( $\dot{V}{CO}_{2}$ ) production were measured on a breath-by-breath basis throughout the running trials. $\dot{V}O_{2}$ and $\dot{V}{CO}_{2}$ during the last minute of the stage were subsequently used to determine substrate utilization using non-protein equations (Jeukendrup and Wallis, 2005), with energy cost being determined as the sum of fat and carbohydrate use when the respiratory exchange ratio was <1. The energy cost was then expressed as J∙kg∙m^-1^ (Fletcher et al., 2009; Shaw et al., 2014). We also expressed running economy as the oxygen cost per kilo per kilometer to allow comparison with previous studies (Robinson et al., 2019; Lepers et al., 2020). Steady state of $\dot{V}O_{2}$ was confirmed visually, and using a linear regression whereby a steady state was defined based on two criteria: the absence of a significant slope in $\dot{V}O_{2}$ (*p* < .05) and a slope in $\dot{V}O_{2}$ of <150 ml min^−1^ (Robergs et al., 2010) (Supplementary File S1, Supplementary Figure S3).

The gas exchange and respiratory compensation points were determined using a combination of methods as detailed by Keir and colleagues (Keir et al., 2022). The gas exchange threshold reflects the highest metabolic rate not associated with metabolic acidosis or disruption of physiological homeostasis, while the respiratory compensation point reflects the highest metabolic rate at which physiological homeostasis can be maintained despite a slight but stable metabolic acidosis (Keir et al., 2022).

Because we observed some hyperventilation during the first minute of the $\dot{V}O_{2}\max$ test (Figure 2), we took extra care to avoid identifying a ‘pseudo gas exchange threshold’ using procedures described by Keir et al. (Keir et al., 2022). $\dot{V}O_{2}\max$ was taken as the highest 30-s $\dot{V}O_{2}$ value. $v\dot{V}O_{2}\max$ was determined by identifying the 30 s over which $\dot{V}O_{2}$ was the highest. If the athlete achieved $\dot{V}O_{2}\max$ during a stage that was not sustained for 1 min, $v\dot{V}O_{2}\max$ was calculated in a pro-rata manner (Sandford et al., 2019; Van Hooren et al., 2022). For example, if the athlete ran only 40 s at the stage where $\dot{V}O_{2}\max$ was achieved, the step increment [0.5 km h^−1^] was multiplied by the percentage of the stage completed (40/60 s = 67%) and added to the speed before the last stage.

### 2.4 Muscle fiber content and mitochondrial function

Two years before the experiments in the current study, the athlete had a muscle biopsy taken from the vastus lateralis as part of other studies registered at clinicaltrials.gov with identifier NCT03666013. Here, we report the athlete’s muscle fiber composition and mitochondrial capacity as previously determined in these studies (Grevendonk et al., 2021; McCrum et al., 2021). Briefly, muscle fiber content was determined using histochemical analysis as described previously (McCrum et al., 2021). Mitochondrial capacity was assessed *ex vivo* using high-resolution respirometry assessed with an Oxygraph (Grevendonk et al., 2021). This approach measures the rate at which mitochondria consume oxygen after different substrate/inhibitor combinations at saturating concentrations are added to the permeabilized muscle fibers in a hyper-oxygenated (∼400 μmol L^−1^ O_2_) respiration chamber. This approach is therefore not limited by oxygen or substrate delivery to the fiber as could be the case *in vivo*. The coupled (state 3) mitochondrial respiration was measured as this provides information on the maximum capacity of the mitochondria to consume oxygen/generate ATP. To this purpose, the following two combinations of substrates and inhibitors were added to the permeabilized muscle fibers: a) 4.0 mmol L^−1^ malate plus 50 μmol L^−1^ octanoylcarnitine and 2 mmol L^−1^ ADP, and b) 4 mmol L^−1^ malate plus 10 mmol L^−1^ glutamate and 2 mmol L^-1^ ADP. Finally, carbonylcyanide-4-(trifluoromethoxy)-phenylhydrazone was added to assess the maximal capacity of the electron transport chain (state 3 uncoupled respiration). Results of these the coupled and uncoupled mitochondrial respiration are presented as the oxygen consumption per mg of wet mass per second. Furthermore, mitochondrial protein complex content was also estimated using OXPHOS protein expression determined from Western blot analysis as described previously (Grevendonk et al., 2021).

## 3 Results

**FIGURE 1:**
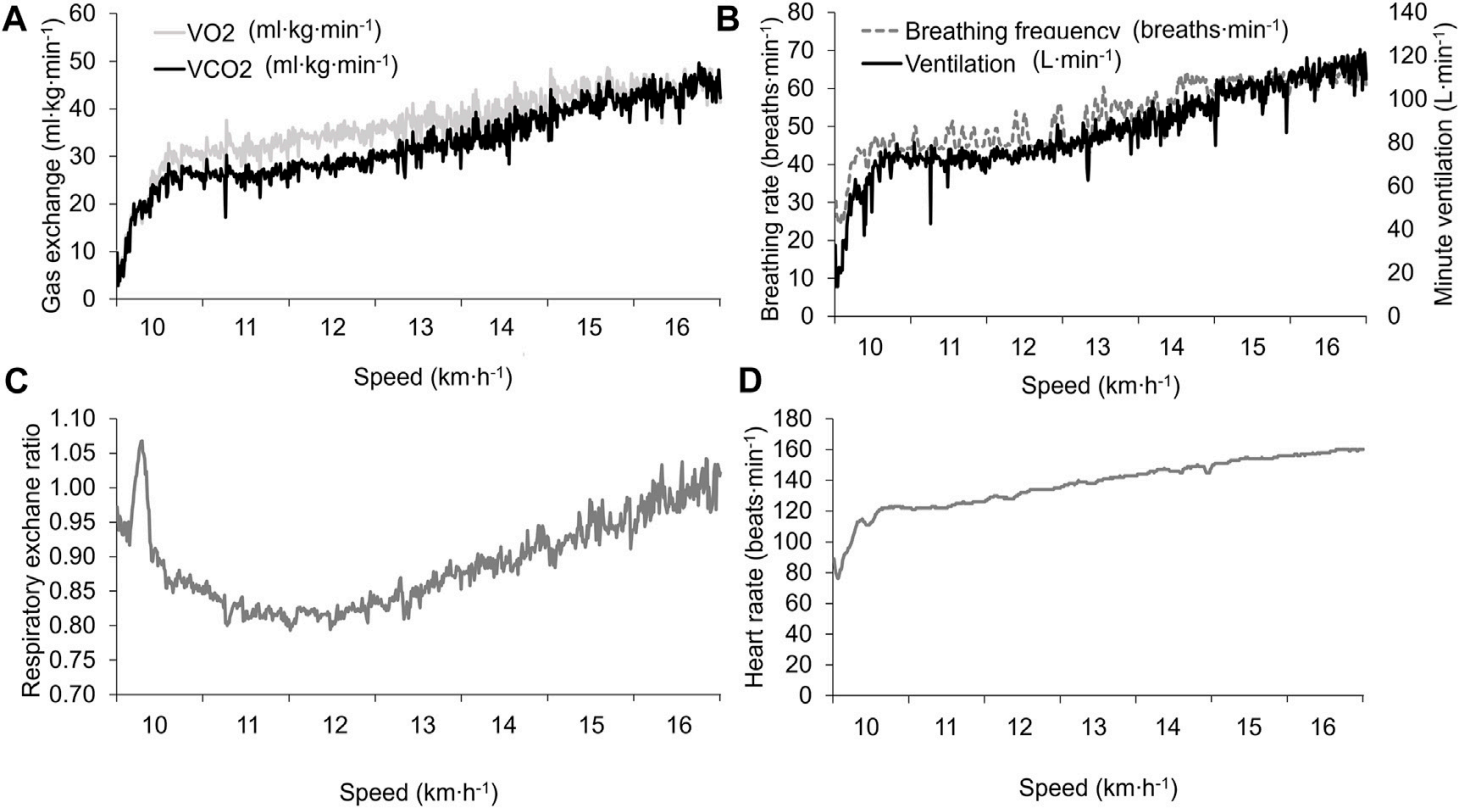
Physiological outcomes during the laboratory vV̇O_2_max assessment. **(A)** V̇O_2_ and V̇CO_2_ relative to body mass; **(B)** respiratory rate and minute ventilation; **(C)** respiratory exchange ratio; and **(D)** heart rate.

Body fat percentage was 13.5%, corresponding to an estimated fat mass of 8.8 kg and fat-free mass of 56.0 kg. $\dot{V}O_{2}\max$ was 46.6 ml kg^−1^∙min^−1^, maximal ventilation was 125 l min^−1^, maximum heart rate was 160 beats∙min^−1,^ and the maximum respiratory exchange ratio towards the end of the exercise was 1.04 (Figure 1; Table 2). $v\dot{V}O_{2}\max$ was reached in the 16 km h^−1^ stage. Because the stage was completed fully, the pro-rata $v\dot{V}O_{2}\max$ was also 16 km h^−1^. The gas exchange threshold and respiratory compensation point occurred at 75.7% (13 km h^−1^) and 93.9% (15 km h^−1^) of the $\dot{V}O_{2}\max$ , respectively.

**TABLE 2:** Anthropometrical and physiological variables of the current and previous world record holder.

Outcome	New record holder	Previous record holder
Anthropometrical		
Body mass (kg)	64.8	64.2
Fat free mass (kg)	56.0	51.9
Physiological		
V̇O_2max_ (ml∙kg^−1^.min^−1^)	46.6	46.9
V̇O_2max_ relative to FFM (ml∙kg^−1^.min^−1^)	53.9	58.0
HR_max_ (beats.min^−1^)	160	156
Oxygen uptake at 12 km h^−1^ (ml∙kg^-1^.km^−1^)	179	187
Oxygen uptake at 13 km h^−1^ (ml∙kg^-1^.km^−1^)	178	192
Oxygen uptake at 14 km h^−1^ (ml∙kg^-1^.km^−1^)	171	188
Oxygen uptake at 15 km h^−1^ (ml∙kg^-1^.km^−1^)	164	176
Oxygen uptake at 16 km h^−1^ (ml∙kg^-1^.km^−1^)	171	n.a
Energy expenditure at 12 km h^−1^ (J∙kg^-1^.m^−1^)	6.63	n.a
Energy expenditure at 13 km h^−1^ (J∙kg^-1^.m^−1^)	3.99	n.a
Energy expenditure at 14 km h^−1^ (J∙kg^-1^.m^−1^)	5.14	n.a
Energy expenditure at 15 km h^−1^ (J∙kg^-1^.m^−1^)	4.35	n.a
Energy expenditure at 16 km h^−1^ (J∙kg^-1^.m^−1^)	n.a., RER>1.0	n.a
Gas exchange threshold (% of V̇O_2max_)	75.7	n.a
Respiratory compensation point (% of V̇O_2max_)^a^	93.9	93

^a^ Note that this was determined using blood lactate in the previous world record holder.

**FIGURE 2:**
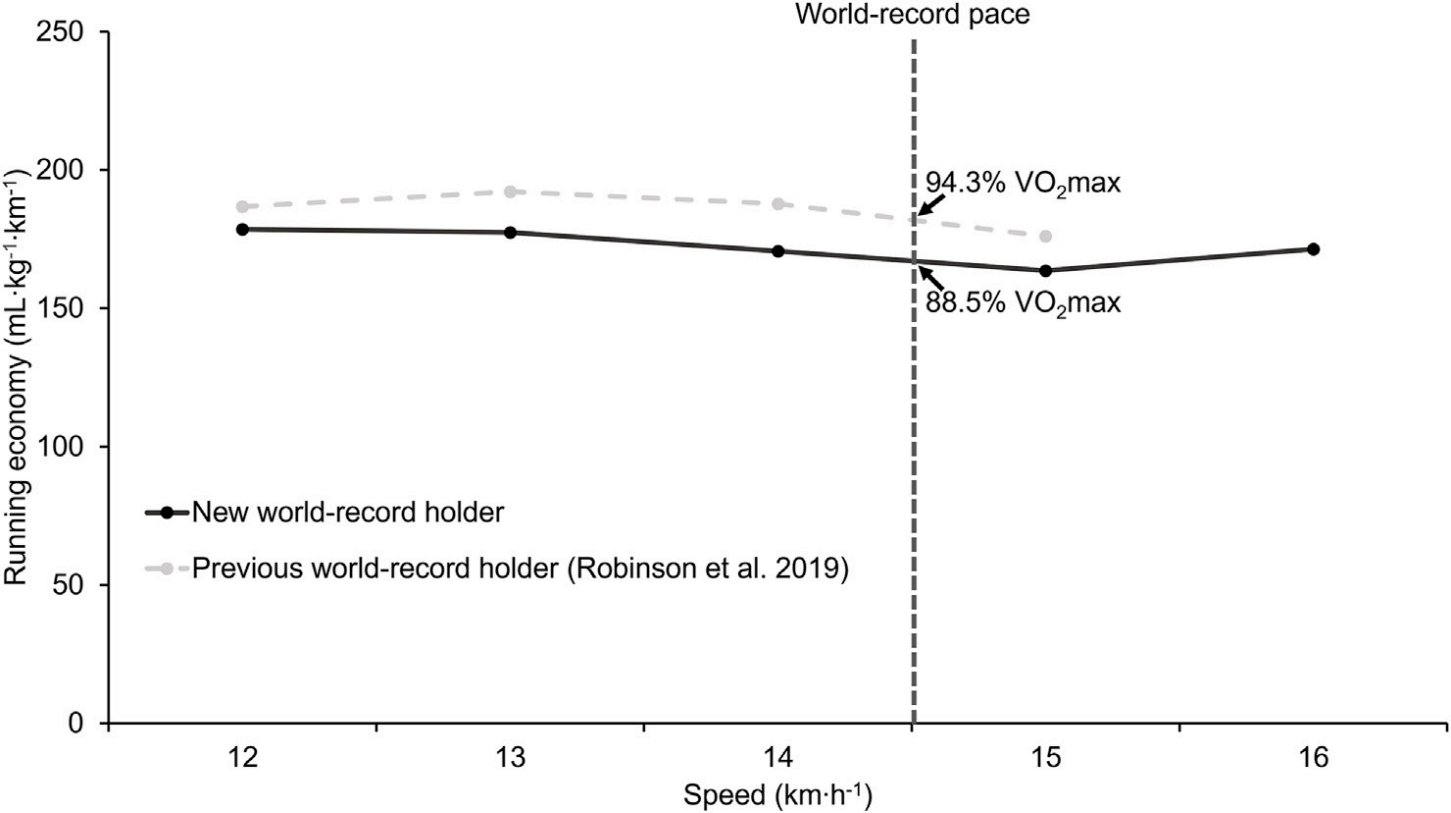
Oxygen uptake at different running speeds for the new and previous world record holders. Data from the previous world-record holder were obtained from Robinson et al. (Robinson et al., 2019) using WebPlotDigitizer. The vertical dashed line represents the world-record running speed of 14.52 km h^−1^.

Oxygen uptake during the steady state for different speeds is depicted in Figure 2 and reported in Table 2. The oxygen uptake of the previous world record holder is also graphed. The predicted oxygen uptake at the world record pace was 41.3 ml kg^−1^∙min^−1^, corresponding to 88.5% of the $\dot{V}O_{2}\max$ . Vastus lateralis fiber content was 90.3% type I and 9.7% type II myosin heavy chain isoform. Mitochondrial respiration rates and content are provided in the supplementary file II (available from https://osf.io/ykdmp). Briefly, *ex vivo* mitochondrial capacity as assessed by the oxygen flux per wet mass was 95.1 pmol s^−1^∙mg^−1^ for coupled respiration, and 115.8 pmol s^−1^∙mg^−1^ for uncoupled respiration.

## 4 Discussion

This study reports the physiological profile of a 71-year-old distance runner who recently (May 2022) broke the world-record marathon for the age category older than 70. The athlete had a very high cardiorespiratory fitness, as indicated by a $\dot{V}O_{2\max}$ of 46.6 ml kg^−1^.min^−1^. This is very similar to the world record holder who broke the record in 2018 (46.9 ml kg^−1^.min^−1^) (Robinson et al., 2019), but lower compared to the world record holder who set the record in 2004 (54 ml kg^−1^.min^−1^ measured at age of 81 years) (New York Times, 2016) or compared to a 75-year-old world-class middle-distance runner (50.5 ml kg^−1^.min^−1^) (Van Hooren et al., 2022). Yet it is considerably higher than untrained individuals of 70 years, where $\dot{V}O_{2}\max$ values are typically ∼20–30 ml kg^−1^∙min^−1^ (American College of Sports Medicine, 2013; Kaminsky et al., 2022). Moreover, the $\dot{V}O_{2}\max$ value of 46.6 ml kg^−1^.min^−1^ would place this athlete in the 60th percentile of 20–29 year-old males according to the 2013 American College of Sports Medicine tables (American College of Sports Medicine, 2013) or in the 50th percentile of the 2022 FRIEND registry (i.e., a database from Nine laboratories in the United States experienced in cardiorespiratory exercise testing with established quality control procedures) (Kaminsky et al., 2022). The relatively high $\dot{V}O_{2}\max$ as compared to individuals of the same age may reflect cardiovascular and muscle tissue adaptations. With regard to muscle tissue adaptations, the athlete’s mitochondrial protein content (i.e., rough proxy of mitochondrial quality) and *ex vivo* respiration parameters were higher than reported for lesser active old males (Grevendonk et al., 2021). Because mitochondrial oxidative capacity shows a strong correlation with body mass specific peak oxygen uptake (van der Zwaard et al., 2021), this finding suggests that these aspects may have partly contributed to the relatively high $\dot{V}O_{2}\max$ this athlete. Additionally, the maximal heart rate (160), is higher than reported in the prior world record holder runner (Table 2) (Robinson et al., 2019) and is also ∼3% higher than predicted for his age using the Tanaka equation (208 - 0.7 x age, which yields 158) (Tanaka et al., 2001), thus likely also contribute to the high $\dot{V}O_{2}\max$ . Finally, the high fat free mass (Table 2) also likely contributed to the high $\dot{V}O_{2}\max$ (Fleg and Lakatta, 1988). Interestingly, when $\dot{V}O_{2}\max$ is expressed relative to fat free body mass for the current and previous world record holder, the new record holder however has a lower $\dot{V}O_{2}\max$ (53.9 vs. 58.0 ml kg^−1^.min^−1^).

Our primary aim was to investigate which physiological variables might have allowed the present master athlete to improve the world record compared to the previous world-record holder. Because his $\dot{V}O_{2}\max$ is very similar, this variable is unlikely to explain the better performance. Instead, the present athlete exhibited a better running economy by approximately 8% at marathon race speed (Figure 2). Further, his gas exchange threshold and respiratory compensation point (i.e., proxy of lactate turn point) also occurred at a relatively high percentage of his $\dot{V}O_{2}\max$ (75.7% and 93.9%, respectively). Although the latter outcomes are difficult to compare with the study on the previous record holder because the authors used lactate to determine the respiratory compensation point (Robinson et al., 2019), our findings indicate that the respiratory compensation point of the present athlete occurred at a slightly higher speed (∼15 km h^−1^ vs. ∼14 km h^−1^), and slightly higher percentage of $\dot{V}O_{2}\max$ (93.9% vs 93%). Yet, the fraction of V̇O_2_max that could be sustained was lower (Figure 2). Using a theoretical approach (di Prampero et al., 1986), it can be estimated that the slightly lower VO_2_max (46.6 vs. 46.9 ml kg^−1^∙min^−1^) and fractional utilization (88.5% vs. 94.3%) of the new world record holder decreased his running speed by 0.09 and 0.01 km h^−1^, respectively. In contrast, his better running economy (170.5 vs. 176.5 ml kg^−1^∙min^−1^) improved his running speed by 0.53 km h^−1^. Therefore, the net beneficial effect corresponds to 0.43 km h^−1^ (Supplementary File S2). This difference is considerably larger than the observed difference (∼0.01 km h^−1^) between the two World records. Differences in other factors such as course, weather, pacing, or shoes could potentially also contribute to the difference between the observed and predicted differences. Nevertheless, these data suggest that further improvements in the World record may be possible. When combining the V̇O_2_max and fraction of V̇O_2_max that can be sustained from the previous world-record holder (Robinson et al., 2019) with the running economy of the current record holder, the predicted world record marathon time for a 70+ year-old athlete would be 2:42:42 h:min:sec (Supplementary File S2). While a combination of all these factors within one athlete is very unlikely, both athletes were not world-class athletes during their younger years and only started to train seriously at a later age. For example, when using a linear regression equation (Supplementary Figure S1) to estimate the marathon performance at the age of 30, the predicted performance would be around 2:25 h:min. Therefore, a performance of <2:45 at 70 years old may be achievable for world-class marathon runners who run below 2:10 at their peak and continue to train at an older age.

The better economy of the new world-record holder, as opposed to the previous record holder, might be explained by aspects such as fiber typology and unmeasured aspects such as mitochondrial efficiency, tendon stiffness, and running technique. Specifically, type I fibers have a higher metabolic efficiency than type II fibers at slow contraction velocities (He et al., 2000), and a high percentage of type I fibers has also been correlated to a better running economy in studies investigating relatively slow running speeds of 12 km h^−1^ (Bosco et al., 1987). Because the current athlete exhibited >90% type I fibers in the vastus lateralis, this fiber distribution may have contributed to his excellent running economy. Indeed, this percentage of type I fibers is substantially higher than the average type I fiber percentage for older adults (e.g., 68% for master cyclists (Pollock et al., 2018) or 41%–45% for sedentary older men (Houmard et al., 1998; McCrum et al., 2021)). Note however that some studies also observed poorer running economy with higher type I fiber percentages at relatively slow running speeds (e.g., 10–11 km h^−1^) (Hunter et al., 2015), thus questioning the overall relevance of fiber type to running economy. As type I fibers may only be more efficient than type II fibers at slow contraction velocities (He et al., 2000), it could be speculated that the current athlete had relatively stiff tendons, which resulted in slow fiber contraction velocities and therefore allowed him to benefit from the higher efficiency of a large percentage of type I fibers. In contrast, individuals with more compliant tendons could show less benefit from a large percentage of type I fibers, especially at higher running speeds. Although this hypothesis requires more research, Achilles tendon stiffness has been shown to be an important factor contributing to running economy (Fletcher et al., 2010). Mechanistically, a stiff Achilles tendon may improve the force-length-velocity potential of muscle fibers, thereby reducing the volume of muscle mass that needs to be recruited to produce force, which in turn reduces energy cost (Bohm et al., 2019). The high Achilles tendon stiffness could in turn be an adaptation to the numerous hills included in most training runs, as uphill running requires a larger force production of the calf muscles, and thus would be expected to lead to larger increases in tendon stiffness. It can be speculated that the lower V̇O_2_max relative to fat free mass of the new world-record holder, as opposed to the previous record holder might also have contributed to the better running economy because previous studies have observed trade-offs between V̇O_2_max and running economy (Fletcher et al., 2009; Shaw et al., 2015; Lannerstrom et al., 2021). The mechanistic explanation for this trade-off is that a higher oxidative phosphorylation associated with a higher V̇O_2_max leads to an increased production of reactive oxygen species (ROS). The larger ROS production can be counteracted by a higher mitochondrial uncoupling (i.e. shunting of protons across the inner mitochondrial membrane, thus decreasing membrane potential and ROS, hereby producing heat instead of ATP), but this process is also associated with a higher oxygen consumption (Hunter et al., 2019). Other findings also indirectly support such a mechanistic trade-off (Larsen et al., 2011; Schiffer et al., 2016). In other words, the lower V̇O_2_max of the new world record holder may have reduced the need for ROS removal and associated higher oxygen consumption by mitochondria during submaximal running, thus also contributing to a better running economy. However, the gross efficiency during cycling of the new world record holder has also been investigated in previous research and was only slightly better than the average gross efficiency in active older athletes (20.8% (Grevendonk et al., 2021)). This collectively suggests that non-metabolic factors such as tendon stiffness and running technique may primarily contribute to the superior running economy.

The physiological adaptations that may contribute to the superior performance of the new world record holder may be a consequence of an almost double training volume of the current athlete compared to the previous record holder (139 vs 72 km wk^−1^, respectively). Indeed, previous studies found associations between training volume and the percentage of type I fibers (Pollock et al., 2018), and larger training volumes have also been associated with smaller age-related reductions in V̇O_2_max (dos Anjos Souza et al., 2023; Burtscher et al., 2022). An important factor contributing to the athlete’s ability to complete such a volume is his resilience to injuries, as he trained every day in 2021 and 2022 leading up to the marathon. The athlete attributed this to performing most of his runs at an easy pace, as guided by the ability to talk easily. Indeed, the average running speed throughout the years (Table 1) and heart rate during three training weeks prior to the world record (Supplementary Figure S4) are lower than his first ventilatory threshold, providing some further support for the generally low intensity of his runs. For example, the athlete spend 97% of his training time in zone 1 (i.e., below the gas exchange threshold; Supplementary Figure S4). Notably, the athlete did rarely perform track interval sessions in an attempt to minimize injury risk. Further, the inclusion of both level and up- and downhill running may have resulted in a better load distribution across tissues, hereby further decreasing injury risk. These three characteristics have previously also been reported in a 75-year-old world-class middle-distance runner (Van Hooren et al., 2022), and suggest that exceptional performance with aging might require high training volumes, but primarily at an easy pace and with variation in inclination to minimize injury risk, thereby allowing athletes to train for years in a row continuously. This continued training might be particularly essential for aging athletes considering the potentially more rapid decrease in physiological parameters with rest in older individuals (Toraman, 2005) and subsequent blunted response to training as compared to younger individuals observed in some (LaRoche et al., 2008) (but not all (Hakkinen et al., 2000)) studies. Such effects might not allow aging athletes to return to high-performance levels after an injury. Mechanistically, type I fibers are less susceptible to muscle damage (Schiaffino and Reggiani, 2011), and the high percentage of type I fibers may have allowed the athlete to complete a high training volume, and hereby reach exception performance at this age. Indeed, whereas most older athletes reduce their training volume with increasing age (Aguiar et al., 2020) to compensate for the reduced recovery ability with aging (Brisswalter and Nosaka, 2013), the current athlete increased in training volume in the last few years (Table 1). This likely slowed down the decrease in performance generally observed with aging (Supplementary Figure S1). His decline in marathon performance between 51 and 71 years old (from 2:41 to 2:54) corresponds to an 8% reduction in running speed in 20 years (4% per decade). This age-related performance decline is lower than the 5%–7% per decade generally observed for well-trained marathoners (Lepers et al., 2020; Lepers et al., 2021). Note that the athlete used short (e.g., 10 min) powernaps between two sessions on the same day, but did not apply periodization, did not follow a special diet, and did not perform any heavy resistance training. Implementing such aspects may further improve the world record in the future.

A limitation of this study is that the athlete had rarely run on a treadmill before, and the running economy might therefore be poorer than overground running economy. However, we provided 8 min of familiarization in line with previous guidelines (Van Hooren et al., 2020) to minimize this effect. Moreover, we compared the heart rate at the 14 and 15 km h^−1^ stages to the heart rate that the athlete recorded while running overground at the same speeds, and the values were approximately similar. Further, differences in treadmill surface stiffness (Colino et al., 2020a; Colino et al., 2020b) and shoe use (Hoogkamer et al., 2018) between the current and previous case study could also contribute to physiological differences. Specifically, the athlete in our study was assessed on the shoes he used during the world record performance, and these shoes contained a carbon plate that may improve running economy (Hébert-Losier and Pamment, 2022). In contrast, the shoes used in the previous studies were standard racing shoes without carbon plates (personal communication Dr. Farquhar 29-7–22). Yet, we used a treadmill that likely exhibited a lower surface stiffness and lower energy restitution (Technogym Exite vs GE T2100) that may have offset some of the beneficial effects of the shoes. Another limitation is that we did not measure lactate concentration and thus approximated the lactate threshold and lactate turn point using gas exchange data. However, several studies showed good agreement between the respiratory compensation point and maximal lactate steady state (Keir et al., 2015; Iannetta et al., 2020; Caen et al., 2021), thus indicating some comparability of these methods. Finally, the muscle biopsy was taken 2 years before the present study, and the athlete has since substantially increased his training volume (Table 1). This may have introduced changes in fiber typology and mitochondrial function. However, we expect this impact to be minor, as the athlete had already been performing endurance training with a weekly training volume of >100 km∙w^−1^ for multiple years before the biopsy. Related to this, mitochondrial respiration shows a high intra-individual variation (∼15% (Jacobs and Lundby, 2021)) and this should therefore be considered when interpreting this data.

## 5 Conclusion

The new world-record holder marathon runner in the men’s 70–74 age category showed a relatively similar V̇O_2_max, a lower fractional utilization but a substantially better running economy than the previous record holder. The better running economy is likely a consequence of an almost double weekly training volume compared to the predecessor and a high percentage of type I fibers. His injury resilience enabled him to train every day for the last ∼1.5 years and achieve international performance in his age group category. The high intensity sustained during the race (here, 88.5% of V̇O_2_max) seems to be a characteristic of elite master marathoners.

## Data Availability

The original contributions presented in the study are included in the article/Supplementary Materials, further inquiries can be directed to the corresponding author.
