# Supplementary material for: A physiological comparison of the new—over 70 years of age—marathon record holder and his predecessor: A case report
Source: Front Physiol. 2023 Feb 13;14:1122315. doi: 10.3389/fphys.2023.1122315 (PMC9969103; doi:10.3389/fphys.2023.1122315)
Supplement: Supplementary file 2 [file DataSheet1.docx]

**Supplemental file I**

*
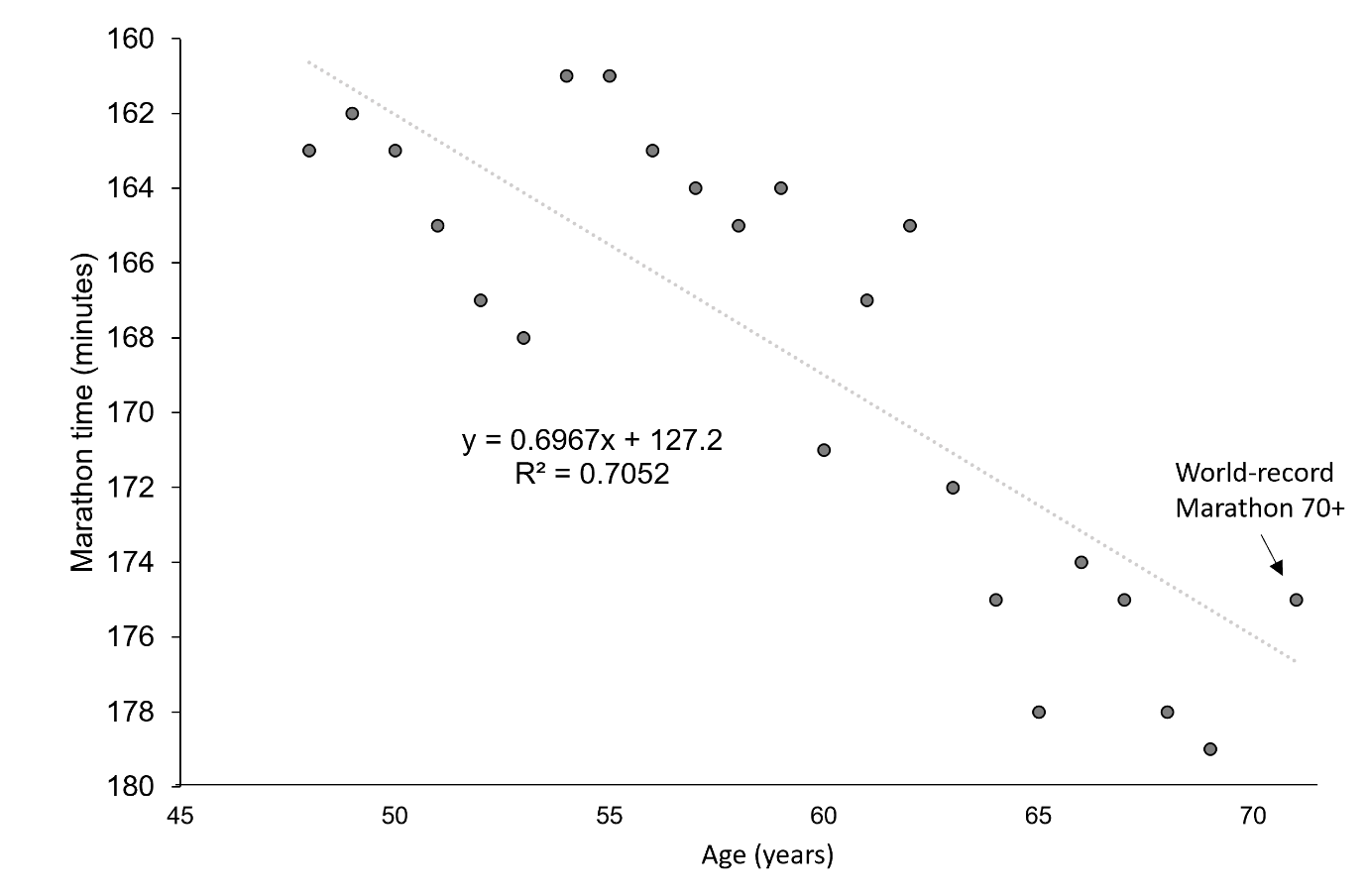
*

**Figure S1.** Marathon best-performance times and a linear regression line depicting the trend over time. Each dot represents the athlete's best marathon performance at each age, as obtained from retrospective data.

**Figure S2.** Marathon times during the Rotterdam marathon from age 42 (1993) to 71 (2022). Note that the athlete started to use shoes with a Carbon plate since he was 70**,** and was sick prior to the race at the age of 64 (time 3:13:36). The dashed line represents a 2^nd^ order polynomial fitted line to depict the trend.


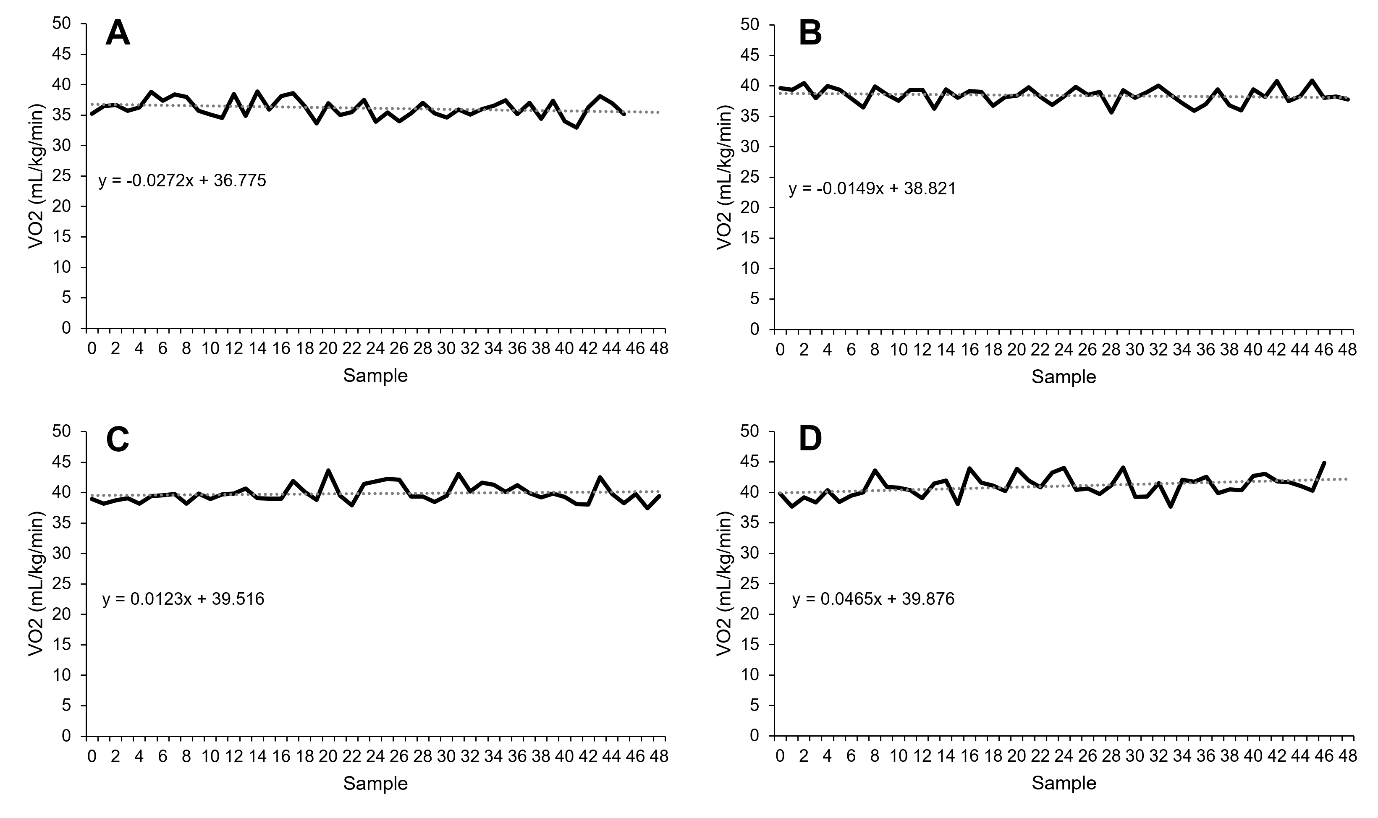


**Figure S3.** Oxygen consumption at 12 (A), 13 (B), 14 (C) and 15 (D) km∙h^-1^, respectively. The dotted line depicts the linear regression line. *P*-values for the slope of the lines were 0.10, 0.26, 0.40, and 0.01 at 12, 13, 14 and 15 km∙h^-1^, respectively. The slope of the line for 15 km∙h^-1^ was 147.6 ml/min and thus below the 150 ml∙min^-1^ threshold set for steady state assessment.

**Training intensity distribution**

The intensity of the training sessions was analyzed based on the time in zone method [1, 2]. To this purpose, three intensity zones were established based on the VO_2_max test results, which were applied to retrospective data obtained from the athlete. Briefly, the athlete exported .Fit files from Garmin Connect from three training weeks in April 2021 (i.e. the month prior to the World record). The heart rate as measured using an optical heart rate monitor (Fenix 6 Pro, Garmin) was used to classify training into the three intensity zones based on the heart rate at the gas exchange threshold (<135 beats.min^-1^, zone 1) and respiratory compensation point (>155 beats.min^-1^, zone 3). The percentage of training time spent in each of the three training zones was then determined for each individual training session, and subsequently averaged per week using a weighted average so that sessions with longer duration had more weight in the computed average. The average and standard deviation of the three weeks were then computed.

**Figure S4**. Training intensity distribution during a typical training week. 97% of the training time was spent in zone 1, 2% in zone 2, and 1% in zone 3. Errors bars represent the standard deviation over the investigated weeks.

**Accuracy optical heart rate monitor**

To verify the accuracy of the time in zones we compared the heart rate measured by the optical heart rate monitor to the heart rate measured with the Polar H10 during the last 60 seconds of the running economy trials, and we compared the highest 30-second rolling average value during the VO_2_max tests (Table S1). Overall, these comparisons show acceptable to good agreement between the two devices, thus providing some confidence in the computed time in zones from the optical heart rate monitor.

**Table S1. Agreement heart rate as measured using two different devices**

| **Condition** | **Mean ± SD (beats∙min^-1^)**  **Fenix 6 Pro (optical)** | **Mean ± SD (beats∙min^-1^)**  **Polar H10** | **Difference(beats∙min^-1^)** |
| --- | --- | --- | --- |
| 12 km∙h^-1^ | 127 ± 1.5 | 126 ± 0.9 | 0.6 |
| 13 km∙h^-1^ | 138 ± 17 | 134 ± 0.7 | 4.6 |
| 14 km∙h^-1^ | 144 ± 0.4 | 143 ± 0.6 | 0.5 |
| 15 km∙h^-1^ | 150 ± 0.9 | 151 ± 0.1 | -0.8 |
| 16 km∙h^-1^ | 152 ± 2.7 | 159 ± 0.4 | -7.4 |
| Peak during VO_2_max | 160 ± 1.5 | 160 ± 0.5 | 0.3 |
| **Overall** | **145** | **146** | **-0.3** |

**References**

1. Seiler KS, Kjerland GØ. Quantifying training intensity distribution in elite endurance athletes: is there evidence for an “optimal” distribution? Scand J Med Sci Sports. 2006;16(1):49-56.

2. Bellinger P, Arnold B, Minahan C. Quantifying the training-intensity distribution in middle-distance runners: the influence of different methods of training-intensity quantification. Int J Sports Physiol Perform. 2020;15(3):319-23.
